# A comparative cross-sectional evaluation of the Field Epidemiology Training Program-Frontline in Ethiopia

**DOI:** 10.1186/s12889-022-13326-2

**Published:** 2022-05-10

**Authors:** Tolcha Kebebew, Tariku Takele, Neima Zeynu, Abraham Muluneh, Medhanye Habtetsion, Jafer Kezali, Sileshi Demelash, Zewdu Assefa, Audrey E. Hu, Mahlet A. Woldetsadik, Reina M. Turcios-Ruiz, Cynthia H. Cassell, Julie Harris, David E. Sugerman

**Affiliations:** 1 Division of Global Health Protection, Center for Global Health, US Centers for Disease Control and Prevention, Addis Ababa, Ethiopia; 2 Center for Public Health Emergency Management, Ethiopian Public Health Institute, Addis Ababa, Ethiopia; 3 Division of Global Health Protection, Center for Global Health, US Centers for Disease Control and Prevention, Atlanta, GA USA

**Keywords:** Field epidemiology training program, FETP, Frontline, Evaluation, Ethiopia

## Abstract

**Background:**

The Field Epidemiology Training Program (FETP)-Frontline is a three-month in-service training aimed at improving surveillance officers’ capacity to collect, analyze, and interpret surveillance data, and respond to health emergencies. We evaluated the effectiveness of the FETP-Frontline which was introduced in Ethiopia in 2016.

**Methods:**

We conducted a comparative, randomized cross-sectional study to assess surveillance-related knowledge, skills, and performance among trained and untrained officers using a structured questionnaire and observation checklist. We compared the knowledge, skills, and performance scores of trained and untrained officers using the Fisher’s Exact test, chi-square test, and t-test at *p*-value < 0.05 for statistical significance.

**Results:**

We conducted the study among 74 trained and 76 untrained surveillance officers. About three-quarters of all participants were male, and the average age was 34 (± 8.6) years. Completeness and timeliness of surveillance reports were significantly higher among trained than untrained surveillance officers. The trained officers were more likely to have produced epidemiologic bulletins (55% vs 33%), conducted active surveillance six months before the survey (88% vs 72%), provided surveillance training (88% vs 65%), conducted strengths, weakness, opportunities, and threats (SWOT) analysis (55% vs 17%), and utilized Microsoft Excel to manage surveillance data (87% vs 47%). We also observed improved surveillance officers’ perceived skills and knowledge, and the availability and quality of surveillance formats and reports among the trained group.

**Conclusions:**

FETP-Frontline trained surveillance officers demonstrated better knowledge, skills, and performance in most surveillance activities compared to the untrained officers. FETP-Frontline can address competency gaps among district surveillance officers in Ethiopia and other countries. Scaling up the program to cover unreached districts can enable achieving the human resource development core capacity requirement of the International Health Regulations 2005.

**Supplementary Information:**

The online version contains supplementary material available at 10.1186/s12889-022-13326-2.

## Introduction

The Field Epidemiology Training Program (FETP) is a globally recognized practice-oriented training that builds the capacity of health professionals in preventing, detecting, and responding to public health emergencies [1–5]. FETP has been adopted by many countries, including Ethiopia [1, 3, 6–12]. The curricula and activities of FETPs are based on the Epidemic Intelligence Service (EIS) training of the US Centers for Disease Control and Prevention (CDC) [13–15], which requires that most parts of training be spent on the field and a smaller amount of time in deductive training. Although FETP has a long history in public health, it was adapted by many countries [4, 5, 16] following the International Health Regulations (IHR) 2005. IHR 2005 and subsequent evaluations of its progress [17] also recommended the training strategy as a tool to fulfil health workforce development, one of the eight IHR core competencies [18, 19].

FETP-Frontline was initiated in African countries following the West African Ebola outbreak of 2014–2016 [20, 21]. It was designed as a three-month in-service training program with the purpose of improving surveillance officers’ capacity to prevent, detect, and respond to public health emergencies. Specific objectives of the training include improving completeness, timeliness, and quality of surveillance data; enabling early detection of diseases; and taking prompt and appropriate public health actions. FETP-Frontline participants spend up to 14 days in three classroom workshops: workshop 1 (6–7 days), workshop 2 (5 days), and workshop 3 (2 days). The remaining 8–10 weeks are dedicated to on-the-job fieldwork to practice, implement, and reinforce what they have learned in the workshops. The classroom lectures are tailored around topics such as surveillance systems and IHR; descriptive statistics and epidemiology; surveillance data quality; data analysis, interpretation and taking actions; monitoring and evaluation; outbreak investigation; linking surveillance and laboratory investigation; analysis of surveillance data quality problems; introduction to Microsoft Excel, Word, and PowerPoint; and scientific communications. Each topic is accompanied by practical group exercises, where participants discuss and share experiences.

**Fig. 1:**
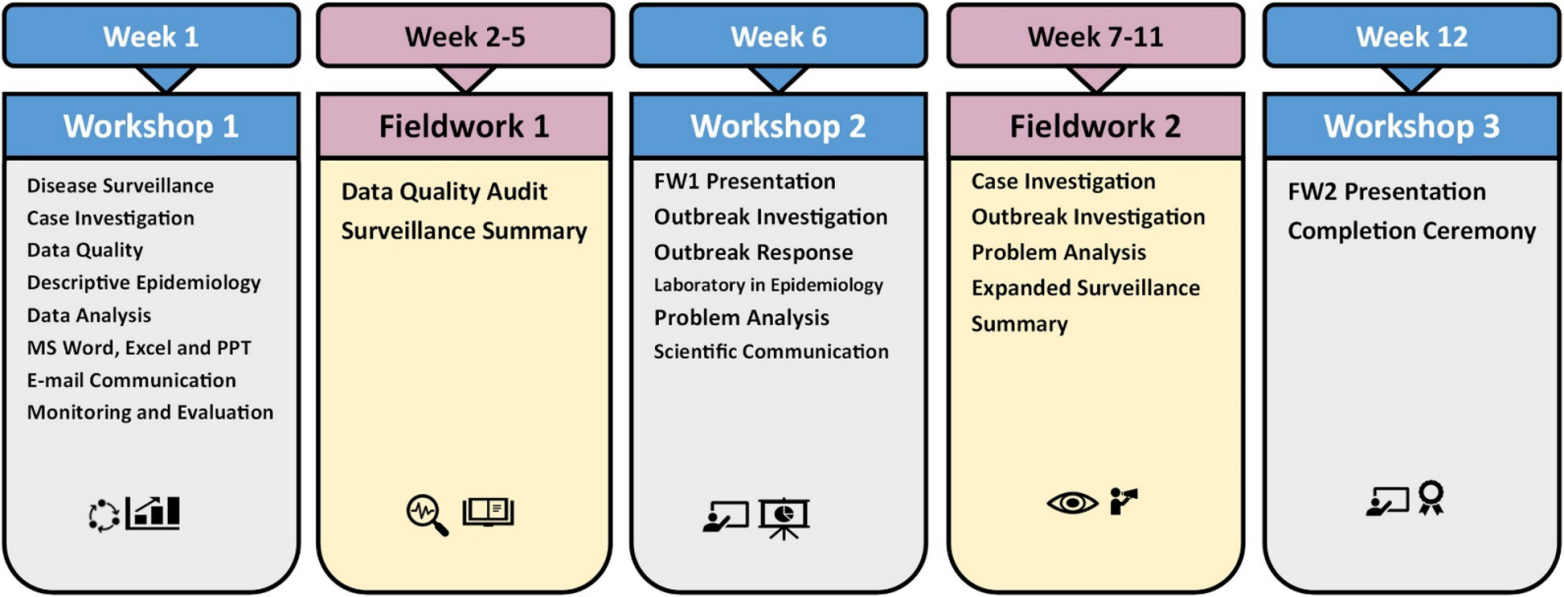
Ethiopia’s curriculum for the Field Epidemiology Training Program-Frontline, [Adapted from the US Centers for Disease Control and Prevention]. Abbreviations – *FW1* Field Work-1, *FW2* Field Work-2

FETP-Frontline was established in Ethiopia in 2016 with support from the CDC, the World Health Organization (WHO), and other stakeholders. The program is hosted by the Ethiopian Public Health Institute’s (EPHI) center for Public Health Emergency Management (PHEM). The curriculum adopted by Ethiopia from the CDC’s FETP-Frontline model [21, 22] is shown in Fig. 1. From December 2017 to March 2019, the program graduated about 250 district surveillance officers, reaching about 25% of the country’s over 1,000 district surveillance officers. Selection criteria to participate in FETP-Frontline include the experience of at least six months as a district surveillance officer and a willingness to serve in the position for at least one year after enrollment into the program. The target of the program was to have at least one FETP-Frontline graduate in all districts of the country.

Despite providing the training to large cohorts, the level of program effectiveness in improving the capacity of surveillance officers was not well documented. This study was aimed at evaluating the impact of FETP-Frontline on surveillance officers’ knowledge, skills, and practices of surveillance and public health emergency response activities.

## Materials and methods

### Study design and setting

We conducted a randomized, comparative cross-sectional study design in September 2019 to evaluate the impact of FETP-Frontline among district surveillance officers in Ethiopia. We conducted the study in 150 districts selected from Addis Ababa City Administration and five regions: Amhara, Afar, Oromia, Southern Nations Nationalities and Peoples’ Region (SNNPR), and Tigray. These regions were selected based on the availably of trained FETP surveillance officers. Using a random number generator, we took a 30% random sample of the districts with FETP-Frontline graduates, which was 74. For comparison, 76 districts without FETP-Frontline enrollees were selected from each of the six locations at 1:1. The inclusion criteria for trained surveillance officers were the completion of the frontline training from December 2017 to March 2019, holding the surveillance officer position during the study, availability during data collection, and willingness to participate in the study. For untrained officers, similar criteria were used, except that they were not enrolled into the training. Untrained officers were selected specifically for each selected trained officer, from a sampling frame of the list of untrained surveillance officers working in the neighboring districts. Officers who were enrolled into the training but had not completed it were excluded. We replaced surveillance officers who were not available at the survey time using the same random procedure.

### Procedure

We developed, pretested, and used a structured questionnaire that covered a wide range of questions meant to assess surveillance officers’ knowledge, skills, and practices. The questionnaire was designed to measure surveillance skills and performance, such as preparing an epidemiologic bulletin or a surveillance summary report; investigating immediately notifiable diseases; conducting health facility supportive supervision; conducting active case searches; doing a strength, weakness, opportunity, and threat (SWOT) analysis; providing formal or informal training; and preparing graphs that show trends of diseases over time. The questionnaire is available in the supplementary materials, named as “Additional file 1.pdf”.

Trained participants were asked, through a face-to-face interview, to self-assess their ability to conduct surveillance activities before and after completing FETP-Frontline, while untrained officers assessed their skills and knowledge at the time of the study. A 5-point Likert Scale, ranging from 1-strongly disagree to 2-disagree, 3-neutral, 4-agree, and 5-strongly agree, was used to assess participants’ perceived surveillance-related knowledge and skills. The quality of selected surveillance reports was assessed using a set of criteria listed on the observation checklist, which was part of the study questionnaire. The authors developed the checklist after reviewing related documents, including the national surveillance guideline [23]. Data collectors, who were public health professionals from the EPHI and the Ethiopia’s Ministry of Health and had extensive experience in conducting surveillance studies, rated the quality of the reports produced by the study participants using the study tool described above.

The study team pilot-tested the questionnaire in May 2019 through 12 one-to-one interviews and five on-site observations. In September 2019, we collected data at district health offices where we conducted interviews in the regional languages: Amharic, Afaan Oromo, and Tigrigna. We managed data and performed analysis using CS Pro 7.3 and STATA 12. We compared knowledge, skills, and practices among district surveillance officers who completed the FETP-Frontline and those not yet enrolled into the training. We calculated frequency distributions, percentages, and average scores and compared it among officers with and without FETP-Frontline training using t-test, Fisher’s Exact test and chi-square test at *p* < 0.05 significance level. We also calculated percentage scores to assess the quality of the reports for each group and identified any differences in knowledge, skills, and practices.

### Ethical considerations

The study was reviewed and approved by CDC’s Project Determination Process and the Institutional Review Board at the EPHI. All participants provided verbal consent and were informed that they had the right to decline participation at any time during the interview.

## Results

### Respondents’ characteristics

We collected data from 150 district surveillance officers, 74 trained and 76 untrained. Almost three-quarters of participants were male and had a bachelor’s degree (Bachelor of Science or Bachelor of Arts). The mean age of all respondents was 34 years (± 8.6), with an average of four years of experience as a surveillance officer. The trained group had a higher proportion of public health officers and environmental health professionals. They also had an additional year of experience as surveillance officers compared to the untrained participants. Two-thirds of all respondents had taken a four-day basic surveillance training, called *PHEM Basic*. However, more FETP-Frontline trained officers had taken the training as compared to the untrained group. Similarly, a significantly higher proportion of FETP-Frontline trainees had received additional training on malaria and vaccine-preventable diseases (Table 1).

**Table 1:** Characteristics of district surveillance officers in Ethiopia, September 2019

**Demographics**	Number		Percent		*P*-value
	FETP-Frontline Trained (*n* = 74)	FETP-Frontline Untrained (*n* = 76)	FETP-Frontline Trained	FETP-Frontline Untrained	
Sex (Male)	56	54	75.7	71.1	0.522^*^
Region					
Oromia	22	22	29.7	28.9	0.998^¥^
Addis Ababa	16	17	21.6	22.4	
Amhara	12	12	16.2	15.8	
SNNPR	9	11	12.2	14.5	
Tigray	8	7	10.8	9.2	
Afar	7	7	9.5	9.2	
Education					
University first degree	62	51	83.8	67.1	0.051^¥^
TVE/Diploma	8	22	10.8	28.9	
Master’s degree	3	2	4.1	2.6	
Other	1	1	1.4	1.3	
Profession					
Nurse	27	42	36.5	55.3	**0.014**^**¥**^
Public Health	28	26	37.8	34.2	
Environmental Health	14	2	18.9	2.6	
Laboratory Technician	1	1	1.4	1.3	
Other	4	5	5.4	6.6	
Types of additional training received					
PHEM basic training	63	37	85.1	48.7	< 0.001^*^
Malaria	49	32	66.2	42.1	< 0.001^*^
Vaccine preventable diseases	55	32	74.3	42.1	0.003^*^

*FETP* Field Epidemiology Training Program, *PHEM* Public Health Emergency Management, *SNNPR* Southern Nations, Nationalities and Peoples’ Region, *TVE* Technical and Vocational Education, Statistically significant results at *p* < 0.05 are shown in bold. *Pearson’s chi-square test; ^¥^ Fisher’s exact test

### Surveillance officers’ performances

The completeness of the weekly surveillance report was higher among the FETP-Frontline trained group (last six months, mean 92%) than the untrained group (last six months, mean 84%) with a statistically significant difference (X^2^ test = 2.16; *p* = 0.016). Similarly, timeliness was significantly higher among FETP-Frontline trained officers (last six months, mean 87%) as compared to untrained officers (last six months, mean 80%), (t-test = 1.82; *p* = 0.035).

**Fig. 2:**
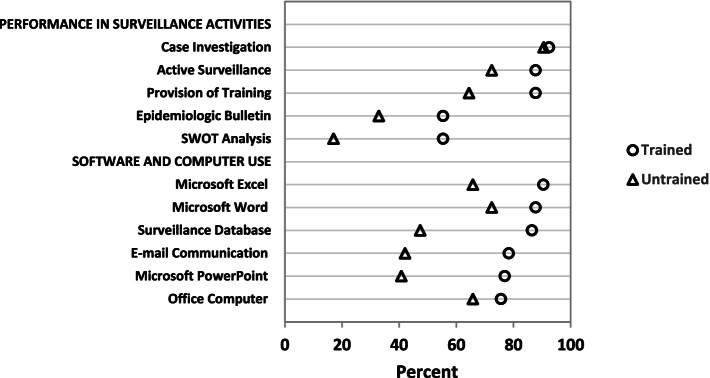
Performance (%) in surveillance activities and computer utilization among FETP-Frontline trained and untrained surveillance officers, September 2019. All differences are statistically significant (*p* < 0.05) except for case investigation, use of office computer and Microsoft Office program

We also observed significantly higher performance in surveillance activities among the trained group as compared to the untrained ones. These include conducting active surveillance, providing training, preparing surveillance summary reports, and conducting SWOT analysis (Fig. 2). Among those who prepared the surveillance summary report, about half prepared it weekly, a quarter prepared it monthly, and the other quarter prepared the bulletin every three months. The surveillance summary report was shared with health facilities, district offices, and zonal surveillance offices. Most reports were shared with multiple offices in hard copy, while a few were disseminated to various offices through social media, including Facebook and Telegram.

A higher number of trained surveillance officers compared to untrained provided training to subordinates in the six months before the survey (88% vs 65%, X^2^ test = 11.22; *p* = 0.001). The surveillance officers had conducted case investigations for most of the immediately notifiable diseases; however, there was no significant difference between the trained (93%) and untrained (91%) groups (X^2^ test = 0.12; *p* = 0.727).

### Use of computer, software for data analysis and reporting

The availability of computers at the participants’ offices was 71% for both groups, with no significant difference (Fig. 2). Similarly, electricity was available about five days per week, on average, for both groups: 4.9 days in a week for the trained and 4.4 days for the untrained groups (t-test = 1.62; *p* = 0.108). However, the utilization of computer programs, specifically Microsoft Word, Excel, and PowerPoint for surveillance activities, was significantly better among the trained group. A significantly higher number of trained officers than the untrained officers developed surveillance databases using Microsoft Excel. In addition, a significantly higher number of trained officers used email compared to untrained officers.

### Perception on surveillance knowledge and skills

**Fig. 3:**
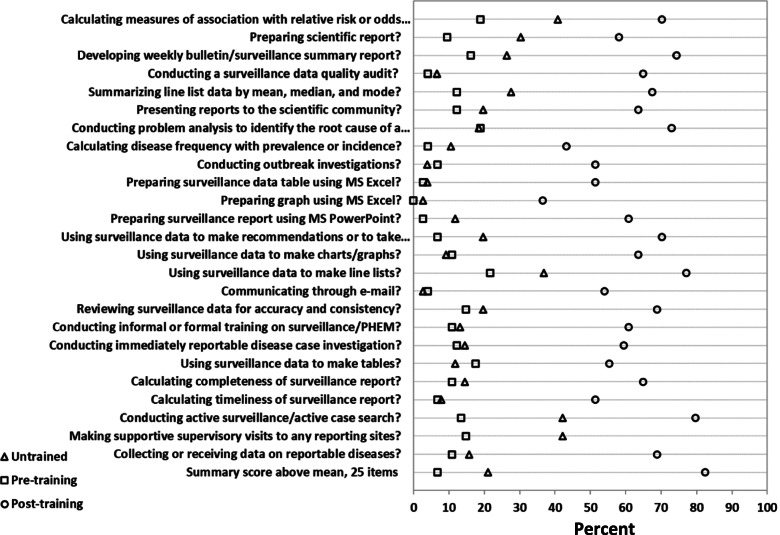
Comparison of perceived knowledge, skills and practices among FETP-Frontline trained and untrained district surveillance officers, September 2019. All comparisons between pre-training and post-training and between untrained and trained are statistically significant (*p* < 0.01). Pre-training and post-training scores are among the trained group, whereas the third score is for the untrained surveillance officers. Self-assessment response ranges are 1: No skill at all, 2: Limited skill, 3: Satisfactory skill, 4: Advanced skill and 5: Expert skill. Responses 4 and 5 were used to compute proportions (in percentage) of those who agreed that they had adequate perceived surveillance knowledge and skills

We measured surveillance officers’ perceived knowledge and skills using the 25 items that assessed respondents’ ability to perform selected surveillance activities. The self-assessed perceived knowledge and skills were higher among trained officers compared to untrained ones, (Fig. 3). We also found that knowledge and skills improved among the trained group after the training compared to their status before enrollment. All the 25 items differed significantly between the groups, both pre- and post-training among the trained officers, and between trained and untrained groups at the survey time, (t-tests; *p* < 0.01).

### Availability of reporting formats

**Fig. 4:**
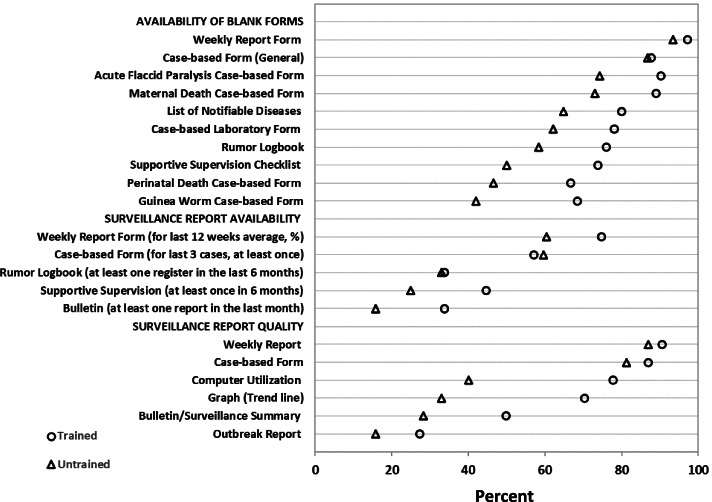
Percent availability and quality of surveillance forms and reports by FETP Frontline training status, September 2019. All differences are statistically significant (*p* < 0.05) except weekly report form, case-based from (general), case-based report, rumor logbook, quality of the weekly report, quality of the case-based report, and quality of outbreak report

The weekly reporting form, used to monitor surveillance data for notifiable diseases, was observed in almost all (95%) workplaces, either in hard copy or soft copy. The availability of weekly reporting form was not significantly different between the trained (97%) and untrained (93%) officers. Similarly, the availability of case-based forms was high (87%) and almost the same among the groups. However, we observed a statistically significant difference in the availability of some other formats: diseases specific case-based forms, case-based laboratory forms, rumor logbooks, the list of notifiable diseases, and supportive supervision checklist (Fig. 4).

### Availability and quality of reports

One of the indicators included in the observation checklist was availability of weekly reports. The proportion of reports available, either in soft copy or hard copy, for 12 weeks before data collection was 75% for the trained group vs 61% for untrained ones (Fig. 4). Availability of surveillance summary and supportive supervision reports were also significantly better among the trained group. However, the availability of complete case-based forms and rumor logbooks were not significantly different among the groups. Although higher quality scores were observed among all the reports of the trained officers, a statistically significant difference was observed in the quality of surveillance summary reports, line graphs, and utilization of computers for surveillance data management and analysis.

## Discussion

This evaluation provided evidence that demonstrated the effectiveness of the FETP-Frontline in improving the knowledge and skills of surveillance officers in Ethiopia. Our study suggested that the capacity of surveillance officers in performing activities, such as collecting data, producing summary reports, conducting active case searches, analyzing data quality problems, conducting supportive supervision, and giving training have been improved.

Most evaluations of FETPs are based on output indicators in which the role of FETP graduates in outbreak detection, investigation, and response was reported [1–3, 6–9, 14, 24–27]. Another means of FETP evaluation is by assessing the number or quality of scientific manuscripts produced by FETP residents and graduates, which are considered indicators of the program’s success [9, 28]. In addition, the involvement of FETP residents in investigations and control of pandemics and public health concerns, including Ebola [29], Zika, malaria, HIV/AIDS, malignancies, diabetes, hypertension, and COVID-19 have been regarded as achievements of the training programs [7, 8, 20, 24, 30, 31]. These studies recommended disease-specific specialty training to equip the workforce with the necessary skill to combat public health emergencies such as the ongoing COVID-19 pandemic [32]. Literature on the evaluation of the effectiveness of the FETP, especially for the FETP-Frontline, is scarce [7, 9, 24, 33]. A few publications reported FETP-Frontline project activities [34–37].

Our study fills the gap in the literature by showing how FETP-Frontline improved graduates’ skills in all areas of the surveillance cycles [21], including data collection, data analysis, interpretation, monitoring, and evaluation. Trained officers showed timely and complete reporting of weekly surveillance data. The trained officers also showed better performance in preparing quality surveillance summary reports. Finding from the evaluation of Kenya’s FETP-Frontline also showed improved on-time reporting and data quality auditing among trained surveillance officers [38]. During FETP-Frontline training, surveillance officers complete the practical exercise of writing up a surveillance summary report, visiting health facilities, and conducting data quality audit, which could have led to better timeliness and completeness of surveillance data. They also practiced how to compute timeliness and completeness of surveillance reports during the training, which could have led to better performances.

Trained officers provided more surveillance training to health facility surveillance focal persons than their untrained counterparts. This could have also led to improvement in many surveillance system components. Trained officers could have also benefitted from gaining skills in scientific presentation and PowerPoint preparation during second and third workshops of the training, which could have improved the overall data summarization and presentation practices.

This study had some limitations. First, trained surveillance officers were more likely to have completed other training, such as the PHEM basic and disease-specific training, than untrained officers. PHEM basic training is a four-day training where major components of the PHEM system (e.g., surveillance, preparedness, response, and recovery) are introduced to newly assigned surveillance officers. Disease-specific training focuses on preparedness, prevention, and control of the outbreaks for vaccine-preventable other diseases, including malaria. The trained group had an additional year of experience, which could have allowed them to attend more training. Therefore, this additional training and experience might have some effect on improving the skills of these officers. Further studies could identify the independent effects of other training on the performance of the surveillance officers. Secondly, this study did not include analyses of challenges [33], such as drop-outs and turnover of trained staff. Thirdly, a higher number of FETP trained participants (84%) were bachelor’s degree holders than the untrained group (67%) which included more diploma holders and nurses. Regional Health Bureau leadership used terminal degree in their selection criteria, in part, because some are expected to join FETP-Advanced, which requires a bachelor's degree. The pre- and post-training self-comparison analysis was incorporated into this study to address these limitations.

## Conclusions

This evaluation identified improved skills, knowledge, and practices among FETP-Frontline trained surveillance officers compared to untrained officers. Improvements were reflected in better timeliness and completeness of surveillance reports, greater knowledge and skills at preparing epidemiological bulletins, conducting active surveillance, providing surveillance training, and conducting SWOT analyses. The trained surveillance officers also had more computer skills, which they used to manage surveillance data and produce graphs and reports. In participants’ self-assessment of perceived surveillance knowledge and skills, trained officers reported higher skills after FETP-Frontline than their skills before the training. Trained officers were also more likely to have surveillance formats and quality reports readily available at their workplaces than untrained group.

FETP-Frontline can be used to build the capacity of district surveillance officers in Ethiopia and perhaps in other countries. We recommend scaling up the program to reach all districts to have at least one FETP-Frontline graduate in each district of the country. The training can help the country fulfil the IHR 2005 workforce development core capacity requirement. Further studies that use longitudinal research design might be beneficial for an in-depth analysis of why and how performance was improved among the trained group and why some of the surveillance components didn’t show any significant differences among trained and untrained groups.

## Supplementary Information

**Additional file 1:** Questionnaire for Woreda PHEM Officers

## Data Availability

Data and other materials can be made available by the corresponding author upon a reasonable request.

## Abbreviations

CDC: US Centers for Disease Control and Prevention
EIS: Epidemic Intelligence Service
EPHI: Ethiopian Public Health Institute
FETP: Field Epidemiology Training Program
IHR: International Health Regulations
PHEM: Public Health Emergency Management
SNNPR: Southern Nations, Nationalities and Peoples’ Region
SWOT: Strength, Weakness, Opportunity, and Threat
WHO: World Health Organization
