# Supplementary material for: A comparative cross-sectional evaluation of the Field Epidemiology Training Program-Frontline in Ethiopia
Source: BMC Public Health. 2022 May 10;22:931. doi: 10.1186/s12889-022-13326-2 (PMC9086414; doi:10.1186/s12889-022-13326-2)
Supplement: Supplementary file 1 — Additional file 1: Questionnaire for Woreda PHEM Officers [file 12889_2022_13326_MOESM1_ESM.pdf]

Questionnaire for Woreda PHEM Officers

Informed Consent

You are being asked to take part in evaluation of the impact of the FETP-Frontline. The purpose of this activity is to improve the methods used to assess the impact of FETP-Frontline training on participants, public health and on the public health surveillance system of the country to improve how FETP-Frontline training will be conducted in the future. You will be asked to complete a survey that takes approximately 40 minutes to complete. There are no right or wrong answers. The survey will be anonymous, and your answers will be kept confidential and will NOT be publicly linked to you. Whenever possible, answers will be presented in aggregate form. You may decline to answer any or all questions and you may terminate your involvement at any time if you choose. There is no direct benefit to you. However, your responses will inform how Frontline training in Ethiopia will be conducted in the future. If you are willing, we will also audio-record the interview not to miss any important information.

If you have questions regarding your rights as a participant in this activity, you can discuss with the person leading this activity: Tolcha Kebebew, the Resident Advisor for FETP-Frontline at +251911450981 or the CDC Resident Advisor for Ethiopia, David Sugerman at +251-9-11-500514. Your participation in this activity is voluntary. It is up to you to decide whether to take part in this activity. If you decide to take part, please confirm your consent in the question below.

Q1. Do you understand and agree to participate in this testing activity?
Yes
No

If No, would you kindly explain why?

Name of interviewer:

Interviewer signature attesting that the participant gave oral consent to participate: ________________

Start Time:

End Time:

Questionnaire ID: /____/____/____/

GENERAL INFORMATION		
	Name	____________________________ (Removed)
1.	What is the name of the woreda/zone/region where you work? (spell out)	Woreda: _____________________<br>Zone: _______________________<br>Region: _____________________
2.	What is your gender?	1. Male<br>2. Female
3.	What is your age in years?	_______ Years
4.	What is your highest educational background?	1. University First Degree (BSc/BA)<br>2. TVET/Diploma<br>3. Master's degree<br>4. Others (specify) _____________________
5.	What is your professional background?	1. Public Health Officer<br>2. Nurse<br>3. Environmental Health<br>4. Laboratory Technician<br>5. Other, (please specify) _____________________
6.	What is your job title now (spell out - do not use acronyms)?	1. PHEM Officer<br>2. Others ___________________________________
7.	In what month and year (Ethiopian Calendar) did you begin the Job with the Title written above?	___________________ Month<br>___________________ Year
8.	How many years of experience in health do you have?	___________________ Years
9.	How long have you worked under your current supervisor (Woreda Health Office Head)?	__________________ Months<br>__________________ Years
10.	Is there a computer or laptop in your office accessible for you?	1. Yes 2. No
11.	Do you have your own computer/laptop?	1. Yes 2. No
12.	Is there mobile network in your Woreda?	1. Yes 2. No
13.	Do you have internet access at your office?	1. Yes 2. No
14.	Do you have your own internet access (e.g. mobile data)	1. Yes 2. No
15.	How many days a week do you have electricity at your office (electric power or generator)?	1. Not at all<br>2. _______ Days
16.	Did you attend trainings on surveillance, reporting, and/or outbreak investigations or any other training related to PHEM?<br>**(Multiple Answers Possible)**	(see sub-table below)
17.	Have you attended FETP-Frontline?	1. Yes<br>2. No **(Skip to Q21)**
18.	When you began FETP-Frontline training, what was your Job Title? (spell out – do not use acronyms)	1. PHEM Officer<br>2. Other (Specify) _________________________
19.	Is your current Job supervisor the same immediate Job Supervisor you had during Frontline training?	1. Yes<br>2. No
20.	In what month and year (Ethiopian Calendar) did you complete or end the frontline training?	________________ Month<br>________________ Year

Question 16:

	Yes	No
a. IDSR/PHEM Basic		
b. VPD		
c. Malaria		
d. Others (specify) _____________		
e. ____________________________		
f.		
g. ____________________________		

21. What is the total number of reporting sites in your woreda?

	Total Number	How many PHEM Focal Persons Received IDSR or PHEM Basic Training?
a. Health Centers		
b. Health Posts		
c. Hospitals		
d. Private Health Facilities (Medium Clinic & Above)		
e. NGO and Others		
Total		

22. For the last 6 months (26 weeks) what was timeliness and completeness of the Woreda?

____________________ % Completeness

____________________ % Timeliness

	**Completeness**		**Timeliness**	
	**Number of reports in the last 1 Months (Out of 4)**	**Number Submitted report in the last week**	**Number of timely reports in the last 1 Months (Out of 4)**	**Number Submitted timely report in the last week**
a. Health Centers				
b. Health Posts				
c. Hospitals				
d. Private Health Facilities (Medium Clinic & Above)				
e. NGO and Others				
Total				

23. Which of the following method(s) do you use to collect or receive surveillance data from health facilities or other reporting sites?

(Multiple Answers Possible)

	Yes	No
a. Paper reports		
b. Telephone calls		
c. SMS/text		
d. E-mail		
e. Other, please list		

24.	Do you develop **bulletin** (or surveillance summary report)?	1. Yes<br>2. No **(Skip to 30)**
25.	How **often** do you develop weekly bulletin (surveillance summary report)?	1. Weekly<br>2. Monthly<br>3. Every three months<br>4. Every six month<br>5. Annually<br>6. I do not develop bulletin/surveillance reports
26.	Have you shared the bulletin to the stakeholders?	1. Yes<br>2. No **(Skip to 30)**
27.	If shared, what are the stakeholders that got the bulletin?<br>**(Multiple Answers Possible; Do not read responses)**	1. Health Centers<br>2. Woreda Health Office<br>3. Other health facilities<br>4. Others ____________________________________

28.	Is there any action taken or recommendations made as a result of the bulletin or summary report?	1. Yes<br>2. No **(Skip to 30)**
29.	If yes, please describe it.	____<br>____<br>____
30.	Have you received data or detected any immediately reportable diseases in the last six months?	1. Yes<br>2. No
31.	How many cases of Immediately notifiable diseases (events) have you reported in the **last six months**?	(see table below)
32.	Have you investigated any of the immediately reportable diseases?	1. Yes<br>2. No
33.	How many of the total immediately reportable diseases have you investigated?	______________ Number of Cases
34.	For how many of the total immediately reportable diseases have you filled **case based reporting form**?	______________ Number of Cases
35.	What activities have you conducted in the case investigations you did?<br>**(Multiple Answers Possible; Do not read responses)**	1. Patient assessment<br>2. Case based form filling<br>3. Lab sample taken/transported<br>4. Feedback given<br>5. others specify ______________________________
36.	Have you visited reporting sites (health centers, etc) to check for data consistency/missing or to conduct data quality audit in **the last 6 months**?	1. Yes<br>2. No **(If no, skip to Q39)**
37.	Is there any action taken or recommendations made as a result of the data consistency check or quality audit?	1. Yes<br>2. No **(If no, skip to Q39)**
38.	If yes, please describe it.	____<br>____<br>____
39.	Have you conducted active surveillance/ active case search at health facility or at community?	1. Yes<br>2. No **(If no, skip to Q44)**
40.	How many supportive supervision visit have you conducted in the last six months? And How many health facilities did you visit?	________________ Number of times<br>________________ Number of health facilities visited

Table for Q31:

	**No of Cases**
a. Measles	
b. AFP	
c. NNT	
d. Rabies	
e. Cholera/AWD	
f. Anthrax	
g. Others ____________________	
h. Others ____________________	
**Total**	

41.	Have provided feedback to the health facilities?	1. On-site feedback<br>2. Written feedback<br>3. No feedback given
42.	Is there any action taken or recommendations made as a result of the supportive supervision?	1. Yes<br>2. No **(If no, skip to Q44)**
43.	If yes, please describe it.	______________________<br>______________________<br>______________________
44.	Have you conducted SWOT analysis?	1. Yes<br>2. No **(If no, skip to Q47)**
45.	Is there any action taken or recommendations made as a result of the SWOT analysis?	1. Yes<br>2. No **(If no, skip to Q47)**
46.	If yes, please describe it.	______________________<br>______________________<br>______________________
47.	Have you provided any formal/informal training to PHEM focal persons?	1. Yes<br>2. No **(If no, skip to Q49)**
48.	How many sessions of the training have you provided in the **last six months**?	____________ Number of format trainings<br>____________ Number of informal trainings
49.	Was there any outbreak/suspected outbreak in the Woreda in the last six months?	1. Yes<br>2. No **(If no, skip to Q57)**
50.	How many of the outbreaks are detected by each of the responsible bodies?<br><br>**Multiple Response Possible**	1. __________ reported from health facilities<br>2. __________ detected by the Woreda PHEM officer<br>3. __________ reported by the community<br>4. __________ other (specify) ______________<br>______________________
51.	How many outbreak/suspected outbreak was reported in the Woreda in the **last six months**?	____________ Number of outbreaks<br>____________ Number of suspected outbreaks
52.	How many of the total outbreak/suspected outbreak was **investigated**?	____________ Number of outbreaks/suspected outbreaks
53.	For how many of the total outbreak/suspected outbreak did you prepare **linelist?**	____________ Number of outbreaks/suspected outbreaks
54.	Have you developed outbreak report for the most recent outbreak?	1. Yes<br>2. No
55.	Have produced line graph for the most recent outbreak?	1. Yes<br>2. No
56.	What was your role in the outbreak investigation and response?<br><br>**(Multiple Answers Possible; Do not read responses)**	1. Leading the investigation and response<br>2. Data compilation<br>3. Active case search<br>4. Developing line list<br>5. Data analysis<br>6. Preparing and sending report<br>7. Other specify ______________________<br>______________________

57.	Have you ever used MS office (Word, Excel, PPT) and other computer program?	1. Yes<br>2. No
58.	Which computer program have you used to analyze/report/present surveillance data?<br><br>**(Multiple Answers Possible; Do not read responses)**	1. MS Word<br>2. MS Excel<br>3. MS PowerPoint<br>4. Internet Explorer<br>5. Other Specify ___________________________________
59.	Have you prepared surveillance data table using MS excel?	1. Yes<br>2. No
60.	Can you use **email** to communicate through internet?	1. Yes<br>2. No
61.	In the last one month, have you communicated through e-mail?	1. Yes<br>2. No
62.	What challenges do you face in receiving surveillance data that is timely, complete, and accurate?<br>(*Probe: issues related to network connectivity, lack of access to a computer, reliable electricity power, challenges related to inadequate training for staff)*	1 ___<br>2 ___<br>3 ___<br>4 ___<br>5 ___<br>6 ___

FETP-PARTICIPANT ONLY

63. Think about the time **before you began FETP-Frontline training** and rate your skill level in performing the following activities according to the scale provided.

1. No Skills at all (Can't perform the activity, no experience)
2. Limited Skills (Perform the activity with major error, few experience)
3. Satisfactory Skills (Perform the activity with some insignificant errors, adequate experience)
4. Advanced skills (Perform the activity with almost no error, good experience)
5. Expert Skills (Perform the activity, excellent experience)

	Response Options				
	**1**	**2**	**3**	**4**	**5**
1. Collecting or receiving data on reportable diseases?					
2. Reviewing surveillance data for accuracy and consistency?					
3. Conducting active surveillance/active case search?					
4. Conducting a surveillance data quality audit?					
5. Using surveillance data to make line lists?					
6. Using surveillance data to make charts/graphs?					
7. Using surveillance data to make tables?					
8. Developing weekly bulletin/surveillance summary report?					
9. Calculating disease frequency with prevalence or incidence?					
10. Summarizing line list data by mean, median and mode?					
11. Calculating measures of association with relative risk or odds ratio?					
12. Conducting problem analysis to identify root cause of a problem?					
13. Using surveillance data to make recommendation or to take action?					
14. Conducting outbreak investigations?					
15. Making supportive supervisory visits to any reporting sites?					
16. Preparing scientific report?					
17. Conducting immediately reportable disease case investigation?					
18. Preparing surveillance data table using MS excel?					
19. Preparing graph using MS excel?					

	20. Communicating through e-mail?					
	21. Preparing surveillance report using MS PPT?					
	22. Presenting report to scientific community?					
	23. Calculating completeness of surveillance report?					
	24. Calculating timeliness of surveillance report?					
	25. Conducting informal or formal training on surveillance/PHEM?					

64. Think about the time **since you completed Frontline training** and rate your skill level in performing the following activities according to the scale provided.

1. No Skills at all (Can't perform the activity, no experience)
2. Limited Skills (Perform the activity with major error, few experience)
3. Satisfactory Skills (Perform the activity with some insignificant errors, adequate experience)
4. Advanced skills (Perform the activity with almost no error, good experience)
5. Expert Skills (Perform the activity, excellent experience)

	**Response Options**				
	**1**	**2**	**3**	**4**	**5**
1. Collecting or receiving data on reportable diseases?					
2. Reviewing surveillance data for accuracy and consistency?					
3. Conducting active surveillance/active case search?					
4. Conducting a surveillance data quality audit?					
5. Using surveillance data to make line lists?					
6. Using surveillance data to make charts/graphs?					
7. Using surveillance data to make tables?					
8. Developing weekly bulletin/surveillance summary report?					
9. Calculating disease frequency with prevalence or incidence?					
10. Summarizing line list data by mean, median and mode?					
11. Calculating measures of association with relative risk or odds ratio?					
12. Conducting problem analysis to identify root cause of a problem?					
13. Using surveillance data to make recommendation or to take action?					
14. Conducting outbreak investigations?					
15. Making supportive supervisory visits to any reporting sites?					
16. Preparing scientific report?					
17. Conducting immediately reportable disease case investigation?					
18. Preparing surveillance data table using MS excel?					
19. Preparing graph using MS excel?					
20. Communicating through e-mail?					
21. Preparing surveillance report using MS PPT?					
22. Presenting report to scientific community?					
23. Calculating completeness of surveillance report?					
24. Calculating timeliness of surveillance report?					
25. Conducting informal or formal training on surveillance/PHEM?					

65.	Since completing FETP-Frontline, have you made any suggestions or action to improve the surveillance system/process?<br><br>(This may include modifying surveillance tools, supporting notification, instituting rumor logs, training staff, auditing data, analyzing data in person, time and place etc.)	1. Yes, please describe your suggestion(s)<br>2. No<br>____________________<br>____________________<br>____________________

66.	If Yes, were any improvements in response to your action or recommendation?	1. Yes, please describe your suggestion(s)<br>2. No<br>____________________<br>____________________<br>____________________
67.	Do you face challenges in using the skills acquired from Frontline training to make changes in the surveillance system?<br><br>(*Probe: No laboratory confirmation, no feedback to/from sites, missing forms, incorrect information records, late reporting, transportation issues, budget issues, lack of access to a computer, reliable electricity power, etc.)*	1. ____________________<br>____________________<br>2. ____________________<br>____________________<br>3. ____________________<br>____________________<br>4. ____________________<br>____________________
68.	Of all the things you learned in Frontline training, is there one that has been the most useful?	1. Yes, please specify which one, and why it has been useful<br>2. No<br>____________________
69.	Do you have any other comments or suggestions for FETP-Frontline?	1 ____________________<br>____________________<br>2 ____________________<br>____________________<br>3 ____________________<br>____________________

CLOSING
Thank you for your time and for answering these questions. If you have any other questions, please let us know

FETP-FRONTLINE NON-PARTICIPANT ONLY

70. Now I am going to ask you about your skill in performing some of surveillance activities, and you are kindly requested to rate your skill level according to the scale provided.

1. **No Skills at all** (Can't perform the activity, no experience)
2. **Limited Skills** (Perform the activity with major error, few experience)
3. **Satisfactory Skills** (Perform the activity with some insignificant errors, adequate experience)
4. **Advanced skills** (Perform the activity with almost no error, good experience)
5. **Expert Skills** (Perform the activity, excellent experience)

	**Response Options**				
	**1**	**2**	**3**	**4**	**5**
1. Collecting or receiving data on reportable diseases?					
2. Reviewing surveillance data for accuracy and consistency?					
3. Conducting active surveillance/active case search?					
4. Conducting a surveillance data quality audit?					
5. Using surveillance data to make line lists?					
6. Using surveillance data to make charts/graphs?					
7. Using surveillance data to make tables?					
8. Developing weekly bulletin/surveillance summary report?					
9. Calculating disease frequency with prevalence or incidence?					
10. Summarizing line list data by mean, median and mode?					
11. Calculating measures of association with relative risk or odds ratio?					
12. Conducting problem analysis to identify root cause of a problem?					
13. Using surveillance data to make recommendation or to take action?					
14. Conducting outbreak investigations?					
15. Making supportive supervisory visits to any reporting sites?					
16. Preparing scientific report?					
17. Conducting immediately reportable disease case investigation?					
18. Preparing surveillance data table using MS excel?					
19. Preparing graph using MS excel?					
20. Communicating through e-mail?					
21. Preparing surveillance report using MS PPT?					
22. Presenting report to scientific community?					
23. Calculating completeness of surveillance report?					
24. Calculating timeliness of surveillance report?					
25. Conducting informal or formal training on surveillance/PHEM?					

CLOSING

Thank you for your time and for answering these questions. If you have any other questions, please let us know

Woreda PHEM Visit Checklist

1. Woreda name:
2. Zone name:
3. Region name:
4. Date of Woreda visit:
5. Data Collector(s) name:

Part I: Availability of surveillance reports

Check for availability of the following reports either as hard copy or soft copy for the last one month or for the last event. Take picture (photo) of the reports.

	Type of surveillance report	Number Available	Comment
6.	Weekly reporting form (Weekly reportable diseases), **(Check for last three months or 12 reports)**		
7.	Case based form (cases of immediately reportable diseases) **(Check for last three Cases)**		
8.	Line list (Outbreak data) **(Check for outbreak in the last six months)**		
9.	Bulletin or surveillance summary report **(Check for last one month)**		
10.	Supportive supervision report or feedback **(Check for the last six months)**		
11.	Outbreak report **(Check for the last six months)**		

Part II: Availability of surveillance formats

Check for availability of the following formats either as hard copy or soft copy.

	Type of surveillance report	Availability		Comment
		Yes	No	
12.	Weekly reporting Form			
13.	Case based form (General)			
14.	Case based form (AFP)			
15.	Case based form (Guinea Worm)			
16.	Case based form (Maternal Death)			
17.	Maternal Death Verbal Autopsy Form			
18.	Maternal Death Facility Based Abstraction Form			
19.	Case based form (Perinatal Death)			
20.	Case based form (Influenza)			
21.	Line list			
22.	Case Based Laboratory Reporting Form			
23.	Daily Epidemic Reporting Form			
24.	List of Notifiable Diseases			
25.	Rumor Log Book (In the comment column write number of rumors/events registered in the last six months)			Number= ________ in the last 6 months
26.	Supportive Supervision Checklist			

Part III: Completeness of Selected Reports

<table>
<tr><th>No</th><th>Item</th><th>Completeness</th><th>Comments</th></tr>
<tr><td>27.</td><td>Bulletin or Surveillance Report:
Check for the most recent weekly bulletin or surveillance report and give response for a-g</td><td>
<table>
<tr><th>Is it complete?</th><th>Yes</th><th>No</th></tr>
<tr><td>a. Woreda background</td><td></td><td></td></tr>
<tr><td>b. Trend of diseases in line graph</td><td></td><td></td></tr>
<tr><td>c. Completeness</td><td></td><td></td></tr>
<tr><td>d. Timeliness</td><td></td><td></td></tr>
<tr><td>e. Explanation of the trend</td><td></td><td></td></tr>
<tr><td>f. Actions taken</td><td></td><td></td></tr>
<tr><td>g. Recommendation</td><td></td><td></td></tr>
</table>
</td><td></td></tr>
<tr><td>28.</td><td>Weekly report forms:
Check for the most recent filled weekly reporting format for the woreda and give response for a-f</td><td>
<table>
<tr><th>Is it complete?</th><th>Yes</th><th>No</th></tr>
<tr><td>a. Region, zone and woreda</td><td></td><td></td></tr>
<tr><td>b. Epi week starting & ending date</td><td></td><td></td></tr>
<tr><td>c. Data filling complete</td><td></td><td></td></tr>
<tr><td>d. HF completeness filled</td><td></td><td></td></tr>
<tr><td>e. Zero report filled</td><td></td><td></td></tr>
<tr><td>f. Name and telephone of reporting official</td><td></td><td></td></tr>
</table>
</td><td></td></tr>
<tr><td>29.</td><td>Case based report forms
Check for the latest filled case based reporting form and give response for a-h</td><td>
<table>
<tr><th>Is it complete?</th><th>Yes</th><th>No</th></tr>
<tr><td>a. Reporting health facility</td><td></td><td></td></tr>
<tr><td>b. Woreda/zone/region</td><td></td><td></td></tr>
<tr><td>c. Specific disease or condition</td><td></td><td></td></tr>
<tr><td>d. Date of onset of the disease</td><td></td><td></td></tr>
<tr><td>e. Age</td><td></td><td></td></tr>
<tr><td>f. Sex</td><td></td><td></td></tr>
<tr><td>g. Outcome</td><td></td><td></td></tr>
<tr><td>h. Lab specimen taken</td><td></td><td></td></tr>
<tr><td>i. Name and telephone of reporting official</td><td></td><td></td></tr>
</table>
</td><td></td></tr>
<tr><td>30.</td><td>Graph Computed from Surveillance Data
Check for the most recent graph produced from summary report of outbreak report and give response for a-d</td><td>
<table>
<tr><th>Is it complete?</th><th>Yes</th><th>No</th></tr>
<tr><td>a. Title (what, when, where)</td><td></td><td></td></tr>
<tr><td>b. X-axis label</td><td></td><td></td></tr>
<tr><td>c. Y-axis label</td><td></td><td></td></tr>
<tr><td>d. Legend (if applicable)</td><td></td><td></td></tr>
</table>
</td><td></td></tr>
<tr><td>31.</td><td>Computer Utilization
Check for availability of the following if there is a computer in use by the PHEM officer</td><td>
<table>
<tr><th>Is it complete?</th><th>Yes</th><th>No</th></tr>
<tr><td>a. MS Excel surveillance data or linelist</td><td></td><td></td></tr>
<tr><td>b. Graphs in MS Excel</td><td></td><td></td></tr>
<tr><td>c. Report in MS Word</td><td></td><td></td></tr>
<tr><td>d. Presentation in MS PPT</td><td></td><td></td></tr>
</table>
</td><td></td></tr>
</table>

<table>
<tr>
<td>32.</td>
<td>Outbreak Report
Check for availability of the following if there is an outbreak report</td>
<td>
<table>
<tr><th>Does the report indicate/discuss?</th><th>Yes</th><th>No</th></tr>
<tr><td>a. Woreda background</td><td></td><td></td></tr>
<tr><td>b. Disease information</td><td></td><td></td></tr>
<tr><td>c. Case definition</td><td></td><td></td></tr>
<tr><td>d. Lab confirmation status</td><td></td><td></td></tr>
<tr><td>e. Descriptive stat (Mean, median, mode)</td><td></td><td></td></tr>
<tr><td>f. Descriptive by Person (Age and Sex)</td><td></td><td></td></tr>
<tr><td>g. Descriptive by Time (days or weeks), onset and end date</td><td></td><td></td></tr>
<tr><td>h. Descriptive by place (Health Facility or Kebele)</td><td></td><td></td></tr>
<tr><td>i. Outbreak curve</td><td></td><td></td></tr>
<tr><td>j. Attack rate</td><td></td><td></td></tr>
<tr><td>k. Measures of association (RR, OR)</td><td></td><td></td></tr>
<tr><td>l. Actions taken</td><td></td><td></td></tr>
<tr><td>m. Recommendations</td><td></td><td></td></tr>
</table>
</td>
<td></td>
</tr>
</table>
